# The International Medical Congress

**Published:** 1881-11

**Authors:** 


### INTERNATIONAL MEDICAL CONGRESS.

#### SECTION ON OBSTETRICS.

*For the Atlanta Medical Register.*

*Oophorectomy—Battey's Operation—Spaying—Castration of Women—Abstract of Remarks of Dr. Battey in Opening, by invitation, the Discussion upon Battey's Operation, at the International Medical Congress, held in London.*—Coming, as I do, from a village so remote and so insignificant in size and importance, as scarcely to be recognized by even a dot upon the map of modern civilization, and deprived, in earlier life, of the adequate mental culture that smooths the pathway to the acquisition of knowledge, I trust I may be allowed to hint, by way of preface, something of the embarrassment with which I appear before this learned body of medical scholars, assembled for deliberation, in the great metropolis of the world.

The operation which has been proposed for this discussion is peculiar, in that it has for its object not the removal of a diseased organ from the body, simply, but the abrogation of a function, in order to do away with its pernicious consequences.

It should be borne in mind that we are not seeking to do an ovariotomy, or an oophorectomy, merely, but to *establish by art the change of life.*

The surgical procedure by which it is sought to produce the artificial change of life has received, from different writers, a variety of designations, no one of which has, as yet, gained the general sanction of the profession.

In America it was first introduced under the name normal-ovariotomy, a name badly chosen and soon abandoned. Spaying was suggested, but generally condemned as inappropriate, repulsive to refined taste, and especially offensive to the subjects of the operation, who have earnestly protested against it. Oophorectomy was considered, and rejected upon the ground that the term had been introduced by Peaslee in his work upon ovarian tumors, and accepted as a synonym of ovariotomy, and, therefore, calculated to produce confusion. The term "Battey's Operation" was offered by Dr. Marion Sims, and is now very generally, though not universally, received. In Germany the operation is known as the "castration of women"—a term as objectionable as spaying.

If the suggestion of Simpson that the ovaries, perhaps, need not be removed, but only ligatured, should be justified by experience, could the operation be properly termed an oophorectomy, a spaying or a castration? Would its essential feature, the production of the change of life by art, be at all altered?

The extirpation of the healthy ovaries, as a remedial measure, seems first to have been contemplated by Dr. James Blundell, of London, as has been lately pointed out by Dr. Aveling. Blundell, however, does not appear to have regarded the suggestion as likely to lead to any practical results, for he remarks that the operation "can scarcely in any instance be necessary."

As early as October, 1865, Battey conceived the idea of producing an artificial menopause for the remedy of disease.

On the 27th July, 1872, Hegar did his first operation, at Freiburg, with fatal result.

August 1st, 1872, Lawson Tait operated, at Birmingham, also with fatal result.

August 17th, 1872, Battey operated, at Rome, Ga., with success, and published his case in the following month. In April, 1873, he defended the operation before the Medical Association of Georgia. He operated again in March, 1874, and a third time in June, 1874, with like success.

December 18th, 1872, Gilmore, of Mobile, Ala., followed Battey in a successful case, which he published in 1874.

August 2d, 1876, Hegar did his second operation, and with success.

*There is a Proper Field for the Operation.*—During the late civil war in America a primipara had a protracted and difficult labor, followed by severe local inflammation, sloughing and occlusion of the whole utero-vaginal canal. Fruitless efforts to restore the outlet were made. The sufferings of the patient became extreme, because of the unrelieved menstrual molimen. The ovaries were removed and fourteen years of perfect anguish came suddenly and completely to an end.

In 1872 Dr. Grange Simons, of South Carolina, reported a very similar case, which died after two years of extreme suffering from unrelieved menstrual molimen, notwithstanding persevering efforts for her relief.

To deny that these were fit cases for the artificial change of life, is but to assert the absurd proposition that years of untold suffering, ending in miserable death, is to be preferred to comfortable health and prolonged life.

*Indications.*—It was foreseen from the very inception of the operation that its sphere of applicability, in exceptional cases, must be as widely extended as the very diverse effects which are known to follow upon preversions of the function of ovulation. Hence, in order to cover the ground fully, and give a key to the selection of suitable cases, it was attempted to mark the boundaries in a comprehensive way by proposing, " ovariotomy to determine the change of life, and the change of life for any grave disease which is incurable without it, and which is curable with it."

More enlarged experience and much study of the subject, have not modified, in any essential particular, the views then entertained upon this point.

Perhaps no safer rule can be laid down to-day by which one may determine, in any given case, the propriety of the operation, than by asking himself three questions, namely:

1st. Is this a grave case ?

2d. Is it incurable by any of the resources of the art short of the change of life ?

3d. Is it curable by the change of life ?

If all three of these questions can be answered affirmatively the case is a proper one, but if not, the operation is not to be justified.

Attempts have been made to group the indications in a more definite way, but it may fairly be questioned whether it is expedient to have an operation so open to abuse go out to the world as a recognized and legitimate remedy for either dysmenorrhœa, menorrhagia or amenorrhœa, however carefully we may qualify the proposition.

That the operation, in its very essence, opens the door for widespread abuse was foreseen from the first, and has been recognized and pointed out by judicious minds everywhere. Its future usefulness to the world depends much upon the jealous care with which the door is guarded. It cannot, therefore, in any case be viewed as an alternative for other means of cure, but must be held, as it was originally offered, for dernier resort.

*Operation.—Mode of Access.*—In America operations have been somewhat divided in choice between the abdominal and vaginal route, both being in use. In Europe the abdominal has been almost exclusively chosen. In a few favorable cases the results obtained by the vaginal method, without any antiseptic precautions, have been all that Mr. Lister, himself, could desire. In view of its rational advantages and the excellency of the results in selected cases, it is believed that it should not be hastily and wholly abandoned, but reserved for such cases only as may, by the free and ready access to the ovaries, be found suitable for it. So frequent, however, are adhesions to be encountered, which can be more satisfactorily dealt with through the abdominal opening, and in cases also where the ovaries are much elevated by the growth of uterine fibromata, laparotomy is to be preferred.

In *dealing with the pedicle* two plans are in vogue. The ligature has to commend it security against hemorrhage, and, when carbolized, comparative innocuousness. It may, however, be objected to as a possible source of local irritation. The ecraseur suggested itself as an efficient substitute, and has been relied upon in at least thirteen operations. Thus far its use has been entirely satisfactory, but a more enlarged experience is needed to establish its safety.

Prof. Simpson has suggested the inquiry whether it be *necessary to remove the ovaries* after strangulating their pedicles with the ligature. Drs. Bardwell and Rumer have essayed to answer the question in a case operated August 9th, 1880, at Mt. Morris, Michigan. Dr. Rumer reports by private letter, "I think the ultimate result may be said to be a perfect success."

It would be interesting to know what will be the clinical history of *diseased* ovaries after strangulation by ligature, and, especially, whether they might be likely to take on malignant disease, as in the case of an undescended, or a dislocated, testis.

*Results.—Mortality.*—The loss of life which has followed upon the operation has been much larger than, *a priori*, might have expected, as will appear from the table of cases collected, which shows a death rate of 18 per cent. But, thanks to the faithful labors of Mr. Lister and his followers, we seem to be upon the eve of greatly improved statistics. Indeed, the late report of Lawson Tait, of twenty-six consecutive cases with but a single death, and the still later list of twenty-six cases, by Savage, without a solitary broken link, testify that the new era is already fairly inaugurated.

*Menses.*—It is a well known fact that double ovariotomy, in exceptional cases, has been followed by continued appearance of the menses. In none of the recorded cases is the evidence of the absence of a third or supplemental ovary, or of a fragment of ovarian stroma hidden in the stump at all conclusive.

These cases, however we may account for them, are admitted to be exceptional, and the rule that the menopause follows the removal of both ovaries is well established by the teachings of experience. But it is not necessary for the success of this operation that the monthly discharge of blood from the uterus should be done away. It is the vascular and nervous purturbation attendant upon the function of ovulation which we are seeking to arrest, and this we can surely accomplish by removing the ovaries. Indeed, it has been shown that very satisfactory results have been obtained in cases wherein the ovaries were but imperfectly removed, and this notwithstanding the continuance of the menses.

In this connection I am able to announce, for the first time, the novel and very extraordinary fact that in one case a small fragment of ovarian stroma, left behind at the operation, not only kept up regular menstruation, but enabled the patient afterwards to bear a child, which is alive to day! *Aphrodisia* is not lost, nor even sensibly impaired by the operation, nor are the *female graces* injuriously affected by it.

*General Health.*—When we consider that the operation is proposed only as a dernier resort, and this in cases of a desperate character wherein life is placed in imminent jeopardy, or existence has become but an intolerable burden, may it not be properly claimed that whatever of beneficial results we may secure are so much actual gain? Is it not hypercritical to object that many of the cases are but partially cured? It is freely admitted that the results are not all we could desire, but are they not all we ought reasonably to demand of a new operation, and in such unfavorable circumstances? May we not expect by accumulated experience greatly to improve upon them in the future?

It is worthy of note that in several instances where the results were unsatisfactory for months (or even a year or more) after the operation, the patients were subsequently greatly improved, and some were even completely cured.

Since the primary object of the operation is to produce the change of life, and the change of life is a gradual process, requiring, sometimes, several years for its full completion, it is premature to set down any case as a failure until ample time has been allowed for the cyclical change to have become complete in its entirety.

